# Interactive OCT-Based Tooth Scan and Reconstruction

**DOI:** 10.3390/s19194234

**Published:** 2019-09-29

**Authors:** Yu-Chi Lai, Jin-Yang Lin, Chih-Yuan Yao, Dong-Yuan Lyu, Shyh-Yuan Lee, Kuo-Wei Chen, I-Yu Chen

**Affiliations:** 1 Department of Computer Science and Information Engineering, National Taiwan University of Science and Technology, Taipei 106, Taiwan; cheeryuchi@gmail.com (Y.-C.L.); darkgrouptw@gmail.com (J.-Y.L.); chen51202@gmail.com (K.-W.C.); karls820210@gmail.com (I.-Y.C.); 2 Department of Dentistry, National Yang-Ming University, Taipei 112, Taiwan; dongyuan.lyu@gmail.com (D.-Y.L.); sylee@ym.edu.tw (S.-Y.L.); 3 Department of Stomatology, Taipei Veterans General Hospital, Taipei 112, Taiwan; 4 Department of Dentistry, Taipei City Hospital, Taipei 103, Taiwan

**Keywords:** OCT-based reconstruction, interactive tooth scanner

## Abstract

Digital dental reconstruction can be a more efficient and effective mechanism for artificial crown construction and period inspection. However, optical methods cannot reconstruct those portions under gums, and X-ray-based methods have high radiation to limit their applied frequency. Optical coherence tomography (OCT) can harmlessly penetrate gums using low-coherence infrared rays, and thus, this work designs an OCT-based framework for dental reconstruction using optical rectification, fast Fourier transform, volumetric boundary detection, and Poisson surface reconstruction to overcome noisy imaging. Additionally, in order to operate in a patient’s mouth, the caliber of the injector is small along with its short penetration depth and effective operation range, and thus, reconstruction requires multiple scans from various directions along with proper alignment. However, flat regions, such as the mesial side of front teeth, may not have enough features for alignment. As a result, we design a scanning order for different types of teeth starting from an area of abundant features for easier alignment while using gyros to track scanned postures for better initial orientations. It is important to provide immediate feedback for each scan, and thus, we accelerate the entire signal processing, boundary detection, and point-cloud alignment using Graphics Processing Units (GPUs) while streamlining the data transfer and GPU computations. Finally, our framework can successfully reconstruct three isolated teeth and a side of one living tooth with comparable precisions against the state-of-art method. Moreover, a user study also verifies the effectiveness of our interactive feedback for efficient and fast clinic scanning.

## 1. Introduction

Because of oral hygiene awareness and advance in dental technologies, modern people generally have dental implants for those root-canal-treated and lost teeth. Currently, dentists would have soft impression material on a slot, would hardly press the slot on the target region, and would later remove it from the target to get the mode while the material gets hard enough. Because this process is long and highly uncomfortable, unconscious movements may happen to cause failures. Furthermore, dentists must visually inspect the extraction to determine whether it captures the desired characteristics. If it is not satisfying, the process repeats. As a result, it is desired to digitally reconstruct the target part for precision, comfortability, reproductivity, and computer-aided examination. Efficient and frequent digital reconstruction may also initiate the possibility of periodic inspection based on newly available artificial intelligence technologies. Therefore, this work aims at developing a digital dental reconstruction framework based on harmless, gum-penetrative optical coherent tomography (OCT).

There are various digital reconstruction methods. Optical reconstruction [1,2,3,4] uses cameras to capture visible light reflected from objects for reconstruction. Additionally, there are also light detection and ranging (LIDAR)systems [5,6,7], which actively emit laser rays and reconstruct objects by stereoscopic matching or reflected time measurement. However, gums occlude part of teeth to limit their reconstruction ability. Furthermore, teeth having complex scattering properties make reconstruction more difficult. In order for volumetric reconstruction, computed tomography (CT) [8,9] uses X rays to penetrate human tissues and to measure the absorption ratio, and magnetic resonance imaging (MRI) [10] uses various magnetic resonances to locate various tissues. However, because they target the whole torso and a tooth only occupies a small portion, the reconstruction may not have enough information for precision. Moreover, they are too noxious for periodic inspection and frequent reconstruction. Optical coherence tomography (OCT) [11,12] can harmlessly penetrate gums using low-coherence infrared rays, but the process easily induces disturbances in its frequency-based reflections resulted in noisy capturing. There are OCT-based dental research for manually inspecting under-gum dental structures [13,14] and periodontal inspection [15,16,17]. However, they do not aim at reconstructing the dental surface and requires manual inspection and examination. To the best of our knowledge, there is still no work applying the technology for dental reconstruction to possibly relieving the time-consuming and painful mode-taking process and to open the possibility for computer-assisted inspection and examination. Additionally, a patient’s mouth has limited operation space for an injector with a small caliber and a short penetration depth and operation range to cover the entire tooth with a single scan. In other words, reconstruction requires multiple scans for the target region. We relieve its noisy nature with a series of processing stages including optical rectification, frequency-to-depth-density transformation, volumetric boundary detection, and surface reconstruction. Additionally, there are flat regions, such as the mesial side of front teeth, to reduce tracking robustness. We enhance aligning robustness by ensuring a proper initial orientation and enough features in the following two manners. First, we design a scanning order for different types of teeth in order to start from a feature-abundant region; second, we have a 3-axis gyro to track the scanning posture in order to have a better initial orientation for alignment. Furthermore, it is important to immediately examine the alignment results to enhance the scanning success rate. Thus, we accelerate the computation with Graphics Processing Units (GPUs) and streamline the data transfer and computations to have an interactive OCT-based dental scanning framework.

Finally, we tested our dental scanner on three isolated teeth and a side of one living tooth with comparable precision against the state-of-art method [4]. Additionally, our system can also reconstruct those parts under gums which the state-of-art fails to achieve. Furthermore, we conduct a usability test to illustrate the importance of visual examination in accelerating the scanning process. Accordingly, we make the following contributions: Optical coherence tomography (OCT) can provide interference responses at the target depth based on its emission frequency for volumetric reconstruction, and thus, we apply this characteristic to develop a tooth gum-penetrative scanner. We overcome the limitations of its noisy imaging, small acquisition caliber, short penetration depth, and short operation range using optical rectification, frequency-to-depth-density transformation, volumetric boundary detection, ordering gyro-assisted point cloud alignment, and Poisson surface reconstruction. Furthermore, it is important to have interactive feedback for examination and to increase the success rate, and thus, we accelerate the rectification, transformation, border detection, and alignment with GPUS and streamline the transfer and processing. As demonstrated in the results, our OCT-based reconstruction framework can properly and efficiently reconstruct teeth while providing intermediate feedback to prevent misses and failure. In other words, this work designs an interactive, harmless, and gum-penetrative OCT-based tooth scanner to precisely reconstruct surfaces for possible mode taking and periodic inspection.

## 2. Overview

**Figure 1:**
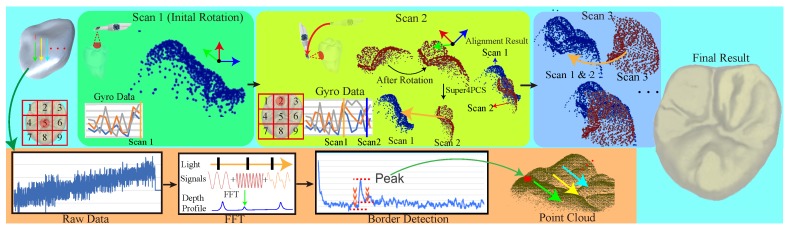
Our scanning process starts at a destined region of a tooth and advances it in a designed order. Each scan consists of injecting an infrared signal of various frequencies, of measuring frequency responses, of collecting depth responses using fast Fourier transform [18], and of volumetrically identifying the boundary. After the first scan, our system initiates the Super 4-point-congruent-set method (4PCS) [19] with the gyro-tracking orientation to align the newly scanned point cloud with the existing for immediate visualization. Finally, we can apply Poisson surface reconstruction [20] for the tooth surface.

Figure 1 illustrates our interactive multiple scanning process and the final surface reconstruction. Our framework applies infrared rays of various frequencies and measures the frequency responses to identify tooth surfaces, which are possibly under gums while covering the entire destination with multiple scans. Before scanning, we first rectify the scanner with our designed device to relieve optical distortions. For each scan, the injector emits infrared rays in a rasterization manner to scan the destined region for 2-D pixel-wise frequency responses. At each pixel, our system first takes the frequency response and uses fast Fourier transform [18] to collect the depth inferences. We then detect the tooth boundary, which has relatively maximal reflection because a tooth is harder than air and gums. However, a tooth surface should be smooth locally, and we then connect these isolated points using simple connecting examination to gain the final point cloud. In order to robustly align multiple scans together, we first ask users to follow a specific scanning order designed for different types of teeth. The process generally starts from the top of the tooth because the region generally contains abundant features to enhance the success rate. Then, we use the attached gyro to track the scanning posture and use it as initial orientation to start Super 4-point-Congruent-Set alignment [19] for better precision and efficiency. After aligning all scanned point clouds, we can finally apply Poisson surface reconstruction [20] to build the tooth surface. In order to provide interactive examination, we streamline the pixel-wise data transfer, FFT transform, and boundary detection while using GPUs to accelerate the processing and alignment. Currently, we cut the whole process into various stages and choose a proper algorithm for each stage. These algorithms can be easily replaced for better precision and efficiency if possible.

## 3. Swept-Source Optical Coherent Tomography

Optical coherence tomography (OCT) is a low-coherence interferometric technique for noninvasive 3-D volumetric imaging with micron-level resolution and millimeters of imaging depth [11]. Generally, the following benefits make it well suited for studying biological structures. (1) It provides real-time subsurface imaging about 1 to 2 mm below the surface at near-microscopic resolution using light backscattered from different layers rather than sound or radio frequency; (2) It requires no preparation at the imaged subjects, and images can be captured non-contact or through a transparent window or membrane; (3) It does not emit ionizing radiation. While imaging, light is broken into two arms: measurement (the interested subject) and reference (a mirror), and reflected light from both arms can be used to estimate the interference pattern at the subject. Areas of the subject at the destined coherent depth reflect a large amount of light to create a greater interference, and any light of which the wavelength is outside the coherent range has almost no interference. This allows us to create a reflectivity profile of the spatial dimensions and location of structures for a special coherent wave length while we can combine multiple depth scans of various wavelengths to construct a cross-sectional tomograph.

Swept source optical coherence tomography (SSOCT) [21] is one of Fourier domain optical coherence tomography (FDOCT) to provide a better depth profile using less scanning time. The cross correlation between the measure and reference arms, which is resultant of the reflected reference intensity from the reference mirror and reflected intensity from the subject [21], is as follows:

(1) $$I^{d}\left(k\right)=\frac{\rho }{2}\left(R^{s}{\left(k\right)}^{0.5}⊗h\left(k\right)\right)$$

where ρ is the detector responsivity, k=2π/λ is a wave number, *h* is the source spectrum, and $R^{s}$ is the reflectivity of the subject. While applying inverse Fourier transform to the above equation with respect to h(k), using fast fourier transformation (FFT) [18], $R^{s}\left(k\right)$ remains as follows:

(2) $$R^{s}\left(k\right)=A_{0}e^{-\sum_{i=1}^{n}M_{i}\mu_{i}^{a}+\sum_{i=1}^{n}N_{i}\mu_{i}^{s}}$$

where *n* is the number of components, $M_{i}$ and $N_{i}$ are combinations of linear coefficients, and $\mu^{a}$ and $\mu^{b}$ are absorption and scattering coefficients, respectively. To summarize, the detected signal would be in the frequency space and we can apply inverse Fourier transform to yield the depth profile [22].

**Figure 2:**
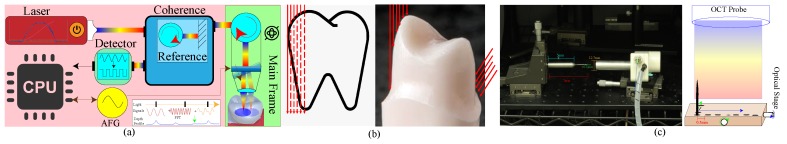
(**a**) Our optical coherent tomography (OCT) device consists of a scanning probe and a main frame while both are connected with an optical fiber. The main frame generates infrared rays of various frequencies, controls the scanning direction, transforms and processes the receiving coherent responses, stitches multiple scans, and reconstructs the tooth while the probe targets rays at the destined region, collects their responses, and tracks the scanning posture. (**b**) Because the injector has a small caliber, it cannot cover the entire tooth. It has a short operation range, and it cannot penetrate the entire tooth as shown in the left. Additionally, the light scatters when it is far from the source to limit its operation range, and thus, the top-down scanning in the right cannot precisely reconstruct the surface and we should scan it from the side. (**c**) The left shows the snapshot of our calibration setting with a manually moving stage and the probe, and the right is the multi-shot calibration scheme, where the needle is placed at designated grid points with a spacing of 0.5 mm.Hardware for Swept-Source Optical Coherence Tomography

In order to have gum-penetrative dental reconstruction, we must assemble an OCT scanner with a set of components for ray emission and coherent response measurement. As shown in Figure 2a, our framework consists of a scanning probe and a main frame. The main frame consists of a control unit of Intel i7-8700 (United States), 16 G memory, Nvidia GTX 1080 Ti (United States), an arbitrary function generator (AFG) of Gwinstek AFG-222 (Taiwan), a swept-source laser injector of Santec HSL-20 50 kHZ (Japan), a coupler, a detector, and an analog-to-digital converter (ADC) of Alazar Tech ATS9350 (Canda). The probe consists of a set of focusing lens, a circulator, and an aiming mirror. While scanning, the swept-source injector first generates an infrared ray of various frequencies and magnitudes. The coupler directs the generated rays to the reference arm and measurement probe. The circulator controls the light direction for emitting and receiving. Our framework uses the signal generated by AFG to control the mirror direction for ray emission and response measurement in a designed rasterization order through the entire field of view in the shape of a square. The detector transforms the returned frequency responses to their corresponding electronic signals using ADC. At the end, the entire device collects frequency responses at a set of 2-D grid points. Additionally, the control unit also streamlines the responses to GPUs, rectifies the capturing, transforms to the depth responses, locates boundary points, stitches various scans, and reconstructs the tooth surface. Because the probe aims at operating in a patient’s mouth, it cannot be too large. Therefore, the effective size of the injector is 6×6 mm${}^{2}$ while the rays comes out in an orthographic manner. Generally, an incisor or canine tooth generally has an averaging size over 9.93×7.81 mm${}^{2}$ while a premolar or molar tooth can be much larger. Although enlarging the injector can possibly solve the problem, it also enlarges the size of the probe and lengthens the operating time, which is about 1.2 s, currently. A longer operating time enlarges the motion possibility to largely reduce the success rate. When discussing with dentists, 1.2 s is roughly the longest acceptable time. OCT has a limited penetration depth of 2 mm, and it is impossible to directly penetrate the entire tooth as shown in the left of Figure 2b. Additionally, in order to ensure good inference signals due to scattering, the effective operation distance is in the range of 0.5 mm to 5 mm. The target region should be entirely inside the effective range for good reconstruction, as shown in the right of Figure 2b. Under these factors, our system must reconstruct the teeth with multiple scans.

### 3.1. Injection and Reception Calibration

Injected rays must pass through a set of lens to emit, and interference rays must pass through another set for possible induction of optical distortions, such as radial and tangential distortions. Therefore, we must calibrate the captured result for precision. Because OCT is sensitive to depth variation instead of color, a traditional black–white checker board does not work. Instead of a shallow–deep checker board, which is hard to construct precisely, we design a needle-based calibration mechanism to have a set of known positions, as shown in Figure 2c. We first have a steel needle with a tip of a length of 2 mm and a thickness of less than 0.1 mm on the top of a 25 × 25 mm${}^{2}$ moving stage of Travel Compact 2-Axis Manual Stage. We fix the probe of which the shooting direction is parallel to the needle and perpendicular to the stage. During the calibration, we choose a set of known locations in the shape of a square grid, of which the spacing is 0.5 mm, and take a shot while having the needle at each location. After designing this calibration device, we remove radial and tangential distortion based on the given set of *N* sampling location, $\left\{\cdots ,\left(S_{i},T_{i},X_{i},Y_{i}\right),\cdots \right\}$, where (S,T) denotes the OCT-captured coordinate and (X,Y) denotes the stage coordinate. The radial and tangential distortions are expressed as the following two equations:

(3) $$\begin{array}{ccc} \left[\begin{array}{c} x\\ y \end{array}\right] & = & \left(1+k_{1}r^{2}+k_{2}+k_{3}r^{4}\right)\left[\begin{array}{c} s\\ t \end{array}\right] \end{array}$$

(4) $$\begin{array}{ccc} \left[\begin{array}{c} x\\ y \end{array}\right] & = & \left[\begin{array}{c} s+2p_{1}t+p_{2}\left(r^{2}+2s^{2}\right)\\ t+2p_{1}\left(r^{2}+2t^{2}\right)+2p_{2}s \end{array}\right] \end{array}$$

where $r=\sqrt{s^{2}+t^{2}}$. The parameters, $\left(k_{1},k_{2},k_{3},p_{1},P_{2}\right)$, are estimated by solving the linear system formed by all samples. However, there may be other distortions and we additionally correct them using thin-plate spline (TPS) interpolation as follows. TPS interpolation creates two as-harmonic-as-possible functions, X(U,V),Y(U,V), based on the given set of *N* sampling location, $\left\{\cdots ,\left(U_{i},V_{i},X_{i},Y_{i}\right),\cdots \right\}$ where (U,V) denotes the corrected coordinate and (X,Y) denotes the stage coordinate. Since our system applies the same interpolation to two coordinate channels independently, the following uses *f* to denote *X* and *Y*. The solution must minimize the bending energy described as follows:

(5) $$I\left(f\right)=\int \int_{\Omega }f_{UU}^{2}+2f_{UV}^{2}+f_{VV}^{2}dUdV$$

and *f* must fulfill the following:

(6) $$f\left(U,V\right)=b_{0}+b_{1}U+b_{2}V+\sum_{i=1}^{N}w_{i}\phi (\|\left(U_{i},V_{i}\right)-\left(U,V\right)\left\|\right)$$

where $\sum_{i=1}^{N}w_{i}=0$, $\sum_{i=1}^{N}w_{i}U_{i}=0$, $\phi \left(r\right)=r^{2}logr$ is the TPS interpolation kernel function, and $\sum_{i=1}^{N}w_{i}V_{i}=0$. This enables the formation of a linear system comprising all of the features, F, to determine the TPS coefficients, $w_{i}$, as follows:

(7) $$\left[\begin{array}{cc} K & P\\ P^{T} & O \end{array}\right]{\left[\bar{W}\;b_{0}\;b_{1}\;b_{2}\right]}^{T}={\left[\bar{H}\;0\;0\;0\right]}^{T}$$

where $K_{ij}=\phi (∥\left(U_{i},V_{i}\right)-\left(U_{j},V_{j}\right)∥)$, the *i*th row of *P* is $\left\{1,U_{i},V_{i}\right\}$, O is a 3×3 zero matrix, $\bar{W}=\left\{w_{1},\cdots ,w_{N}\right\}$, and $\bar{H}=\left\{F_{1},\cdots ,F_{N}\right\}$ is the list of the sampling $\bar{X}$ or $\bar{Y}$. After solving Equation (7), the proposed system respectively uses $\bar{W}$, $b_{0}$, $b_{1}$, and $b_{2}$ for two coordinate channels in estimating its true world coordinate.

### 3.2. Three-Axis Posture Tracking Gyro

Point-cloud alignment generally requires solving an optimization problem, and it is important to have a proper initial orientation. In other words, to have a proper initial orientation can accelerate the alignment process. Thus, as shown in Figure 2, we append the probe with a 9-axis accelerometer-gyroscope-magnetometer unit consisting of a STMicroelectronics L3GD20 gyroscope, a STMicroelectronics LIS3DH accelerometer, and a STMicroelectronics LSM303DLHC magnetometer. Although it can both track the position and orientation, the positioning precision is generally within a range of a few cm and it only deteriorates registration according to our experiments. As a result, this work only uses the attached gyro to have the initial orientation for alignment as discussed later.

## 4. Interactive Dental Scanning

OCT imaging is considered the optical analog to ultrasound, but it can achieve higher resolution through the use of near infrared wavelengths at the cost of decreased penetration depth for noisy capture and of having a smaller field of view. Thus, in order to precisely reconstruct teeth, we must first robustly and coherently determine the dental surface points and, then, our system must align multiple point clouds to cover the entire target portion. Additionally, operators should also be able to examine the immediate results to ensure the scanning success, and thus, we accelerate the whole process with GPUs and streamline data transfer and computations for interactivity.

### 4.1. Boundary Detection

Generally, inferences inside homogeneous material have similar intensities but vary largely while moving across a boundary. Therefore, we examine the variation along the depth and find points with local maximal variation to identify tooth boundaries. Those points at which variation magnitude is larger than $T^{top}$ and smaller than $T^{bottom}$ are eliminated because they have a high chance of being induced by lens reflection and signal noises. This work sets $T^{top}$ = 32,768 and $T^{bottom}$ = 39,441 for all experiments. We process all scanning rays to get all boundary candidates, but noises still can induce outliners in the detection. Because boundaries should be spatially continuous, we randomly select several candidates as centers and label them as boundary pieces. For those neighboring candidates of boundary pieces, if its distance to the projected plane of neighboring labelled boundary pieces is shorter than $T^{distance}$, where we set it to be 1.0 for all experiments, we add it into the nearest pieces. Otherwise, we remove it. The process finishes while there are no candidates left. We can gain a set of points for the targeted smoother and continuous tooth surface.

### 4.2. Parallelize Point-Cloud Alignment

Multiple scans are necessary to overcome the limitations of a small caliber, a short penetration depth, and a short operation range. As a result, we must align the newly scanned point cloud with the existing one. In other words, given the source and target point clouds, $P^{S}$ and $P^{T}$, we must determine their relative rigid transform, T. Currently, we choose the Super 4-point-congruent-set method (4PCS) [19] because of its robustness and possible GPU acceleration. At the beginning, the 4-point-congruent-set method picks up a planar 4-point basis from $P^{S}$ and find its congruent 4-point set in $P^{T}$. Then, we can estimate T based on these two sets and its matching error using the iterative closest point method as follows:

(8) $$E\left(T\right)=\sum_{i=1}^{n^{S}}\sum_{j=1}^{n^{T}}M_{i,j}{∥{\bar{P}}_{i}^{S}-{\bar{P}}_{j}^{T}∥}^{2}$$

where $M_{i,j}$ is the matching function and $W_{i,j}=1$ if ${\bar{P}}_{i}^{S}$ and ${\bar{P}}_{j}^{T}$ are a matching pair. We can repeatedly choose another planar 4-point basis and its congruent 4-point set in the manner of RANdom SAmple Consensus (RANSAC) until it converges or uses up the computation time. The Super 4-point-congruent-set method [19] accelerates the congruent set selection process using a sphere rasterization and a kd-tree data structure. Although the method can theoretically start from any relative transformation, this work requires a fast converging process while using less samples from both clouds. Under this constraint, it is better to start with better initial transformation, $T^{in}=R^{in}+{\bar{t}}^{in}$. Therefore, we use the attached gyro to have a better initial orientation $R^{in}$. Additionally, we estimate the rotation center of the scanning probe. First, we determine the centroid and major extension plane of the source cloud using principal component analysis (PCA). Then, we compute a fitting sphere with the following criteria. Its center should be on the line passing through the centroid and parallel to the plane normal. Its radius should be the average size of the tooth. Finally, we use the rotation center, and the orientation to determine its initial translation ${\bar{t}}^{in}$.

### 4.3. Effective Scanning Order

**Figure 3:**
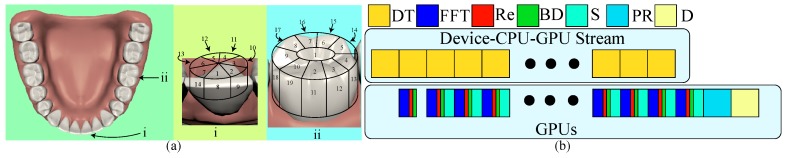
(**a**) This illustrates our two designed scanning orders based on the size of its occlusal surface where the left shows the location and viewing direction of two representative teeth; the middle is for the one of a small occlusal surface, and the right is for the one of a large surface. (**b**) There are two parallel streams: data transfer and GPU computation. While transferring a slice of scanning data (DT), GPUs also compute fast Fourier transform (FFT), optical rectification (Re), and boundary detection (BD). At the end of scanning, GPUs finalize the last slice, do the alignment, and show the stitched point cloud.Effective and robust scanning ordering

Although the gyro’s initial orientation can enhance the robustness of registration, certain dental regions are flat and lack alignment features. It is better to start scanning from feature-abundant regions due to the possibility of having enough tracking features. Figure 3a shows our two different designed scanning sequences based on the size of its occlusal surface. First, if the tooth has a large occlusal surface such as a premolar and a molar tooth, we should start from the center of its occlusal surface because this region contains highly geometric details. Then, we scan the entire occlusal surface counterclockwise until it covers the top of the side surface. Finally, we move down layer-by-layer on the side surface until it covers the entire tooth. Second, the tooth with no or a small occlusal surface, such as an incisor tooth, has a feature-abundant incisal edge, and thus, we start scanning from the top side of the edge. Similarly, we then scan the top of the side surface counterclockwise and extend the scanning layer-by-layer to cover the entire tooth.

### 4.4. Streamlined Data Transfer and Computation

In order to interactively examine each scanning result, our framework must be able to provide immediate visualization of the aligned point cloud after each scan. We first let GPUs accelerate fast Fourier transform (FFT), optical rectification (Re), boundary detection (BD), and point-cloud alignment. Additionally, data transfer generally takes the longest time. It would be better to segment the whole volumetric data into slices, where a slice consists of those scanning data in which the *y* coordinate is the same for data transfer while FFT, Re, and BD can be executed in GPUs parallel to the transfer as shown in Figure 3b. At the end, GPUs can finalize the processing computation, align the point clouds, and visualize the result.

## 5. Surface Reconstruction

After scanning, we have a set of surface points, $P=\{{\bar{P}}_{1},\cdots ,{\bar{P}}_{n}\}$. However, a point cloud is unstructured and hard for possible geometric operations such as smoothing and editing. Therefore, our framework reconstructs the surface using Poisson surface reconstruction [20], which considers all the points at once to have high adaptation for various geometries and topologies and is highly resilient against noise. We would like to reconstruct the surface, S, by having an indicator function of the following shape:

(9) $$\chi^{S}\left(\bar{p}\right)=\left\{\begin{array}{cc} 1 & \mathrm{if}\;\bar{p}\in S\\ 0 & \mathrm{otherwise}. \end{array}\right.$$

Then, we can minimize the deviation between the gradient of the indication function and approximate orientation function of these points, $\vec{V}$ as $\min_{\chi }∥\nabla \chi -V∥$. While applying the divergence operator, we have a Poisson problem as ∇×∇χ=∇×V⇔▵χ=∇×V. When solving it discretely using an adaptive octree method [20], we can reconstruct the scanned tooth surface.

## 6. Results

Our OCT-based scanner can penetrate gums to reconstruct teeth. We first used it to reconstruct a living incisor and three isolated tooth for analysis of its operational effectiveness. Later, we also showed a usability test by using our interactive framework to scan an incisor, premolar, and molar to verify its usefulness.

### 6.1. Ablation Study

**Figure 4:**
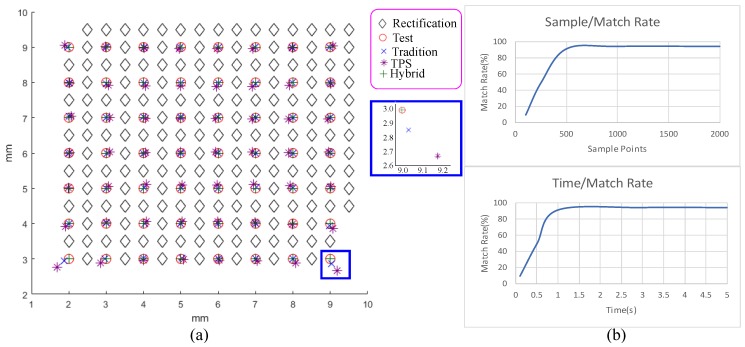
(**a**) This shows the rectification results of traditional radial and tangential distortion removal (blue crosses), thin-plate spline (TPS) interpolation (purple stars), hybrid of both (green pluses), and ground truth (red circles). Their averaging errors are as $6.96\times 10^{-2}$, $2.58\times 10^{-2}$, and $1.52\times 10^{-2}$ mm, and their maximal deviations are $3.95\times 10^{-1}$, $1.44\times 10^{-1}$, and $7.67\times 10^{-2}$ mm. (**b**) This shows the relation between the alignment success rate and the number of samples (top) and computation time (bottom).Rectification analysis

Rectification is important for the reconstruction precision, but it is different from board-based ones, while other stages have been comparatively analyzed in the past research. Thus, we conducted a simple experiment to analyze our rectification error. First, we chose a set of samples distributed regularly inside a rectangle of 8×8 mm${}^{2}$ with a spacing of roughly 0.5 mm and had their position as ground truths marked as black circles and cyan diamonds in Figure 4a. We use half of the samples marked as black diamonds for calibrations, and the other half marked as red circles following the capturing process as rectification to capture these samples and to rectify them back to the corrected positions using the radial and tangential correction, TPS interpolation, and hybrid of both marked with blue crosses, purple stars, and green pluses. The average deviations were $6.96\times 10^{-2}$, $2.58\times 10^{-2}$, and $1.52\times 10^{-2}$ mm, and the maximal deviations were $3.95\times 10^{-1}$, $1.44\times 10^{-1}$, and $7.67\times 10^{-2}$ mm. The hybrid performs better than the other two acting alone.

The allowed computation time and the number of points selected from a cloud can largely affect the alignment success rate. Our interactive mechanism requires less computation time but still maintains a high success rate. Thus, we determined these parameters as follows. First, while discussing with dentists, they suggest that 90% should be fine for a success rate, and it is fine to wait 2 to 3 s after scanning. Therefore, we target a success rate around 90% and an alignment computation time below 2 s. Second, we chose two point clouds which are hard to align and randomly selected $N^{sample}$ samples from both point clouds to attempt aligning them 100 times while using only 2 s of alignment time as shown in the left of Figure 4b. Based on the result, the number of samples is chosen to be 500. Third, we tested aligning the same two point clouds while using 500 samples and various computation times as shown in the right of Figure 4b. Accordingly, we choose the execution time to be 1 second, which can achieve the targeting goal.

Our framework only uses a simple thresholding-and-connecting method to detect tooth boundary. In order to evaluate its effectiveness, we have conducted a simple comparison against Grabcut [23], a commonly used segmentation algorithm. First, we adapted the source code of Grabcut from the OpenCV library into our framework and used their default setting to identify the tooth boundaries of each capture data slice. Then, we used the identified boundary points to reconstruct the tooth surface. Finally, we measured the average and maximal deviations against the ground truth of the corresponding tooth. As shown, our method has respective average and maximal deviations of $4.07\times 10^{-2}$, $4.30\times 10^{-2}$, and $5.25\times 10^{-2}$ and of 1.24, 1.15, and $5.68\times 10^{-1}$ mm, for incisor, premolar, and molar, respectively, while Grabcut has them of $8.75\times 10^{-2}$, $8.79\times 10^{-2}$, and $1.27\times 10^{-2}$ and of 2.26, 1.58, and 4.45 mm, respectively. This shows that our algorithm can perform better against naive Grabcut while it is more efficient and easy to accelerate with GPUs.

### 6.2. Reconstruction Precision and Efficiency

**Figure 5:**
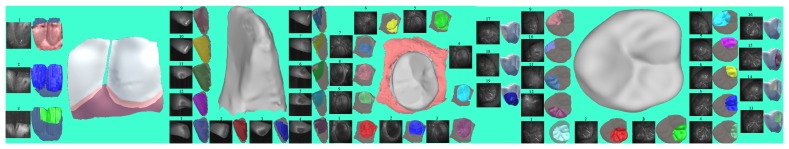
This shows the sequential reconstructed results of a side of a living incisor, an isolated incisor, an isolated premolar, and an isolated molar along with their depth responses in the designed scanning order, where green marks newly added points and blue marks the already existing points.Reconstructed point clouds of teeth during the scanning process

**Figure 6:**
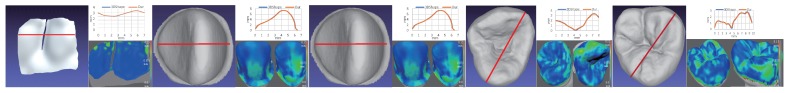
This illustrates the precision analysis when using the model constructed by the state-of-art digital intraoral scanner Carestream’s CS 3600 [4] as ground truth for the living incisor and the isolated incisor, premolar, and molar teeth, where the average deviations are 8.71, 27.3, 28.4, and 30.6 μm, respectively, and the maximal are 38.3, 263, 135, and 235 μm, respectively. The **left** shows the reconstruction surface; the **right top** shows the visualization of height differences at the cutting line, where blue marks the results reconstructed by the state-of-art, and red marks those by ours; and the **right bottom** visualizes the reconstruction errors while comparing to the model generated by the state-of-art using the rainbow coloring scheme.Precision comparison

In order to analyse our reconstruction precision, we first used our OCT scanner to reconstruct a side of a living incisor and isolated incisor, premolar, and molar teeth using our proposed scanning order incorporated with the gyro as shown in Figure 5. We applied the state-of-art digital intraoral scanner of Carestream’s CS 3600 [4], targeting at the same teeth for ground truths. We can estimate the deviation of ours and the ground truth and visualize it on the surface using the rainbow color scheme. As shown in Figure 6, the central part generally has lower reconstruction errors but the exterior part has larger reconstruction errors. The reconstruction results have averaging deviations of 8.71, 27.3, 28.4, and 30.6μm and maximal deviations of 38.3, 263, 135, and 235 μm, respectively.

**Figure 7:**
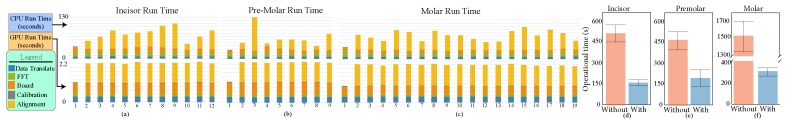
(**a**–**c**) The run time of each stage at various scans using a CPU (the top) and a GPU (the bottom) for the incisor, premolar, and molar teeth and (**d**–**f**) the statistics of our user study in the time of operation in the unit of seconds with/without using the visualization system.Run time analysis

At the same time, we also analyse the computation time of data transfer, FFT transform, border detection, and point cloud registration for each scan using the Computing Processing Unit (CPU) and GPU, as shown in Figure 7a–c, using a computer with Nvidia GTX 560 Ti, Intel i7 3770, and 16 GB main memory. As shown, the data transfer generally takes about 1.2 s while rectification, FFT, and border detection take less than that. We can combine these together to have an average of 1.2 s for these three operations.

### 6.3. Robustness of Scanning Ordering and Gyros

In order to understand the effect of our scanning order and incorporation with the gyro, we use the ordering data of molar scanning along with the gyro information to conduct the following experiment. We first scanned the target 11 times using our designed order incorporated with the gyro-tracking orientation without visualization to examine the success rate of the reconstruction. Totally, we got 10 successful results and had a success rate of 90.9%. Then, we conducted two different sets of analyses on these data sets. The first analysis aligned ordering scanning point clouds using the Super 4PCS algorithm without initiating its orientation using the gyro. We selected a scan from the scanning sequence and registered it to the assembly point cloud constructed by aligning all previously scanned point clouds. We ran this test 150 times and recorded its success rate as 32.3%. The second analysis intended to register two randomly selecting neighboring point clouds while initiating the registration orientation using the gyro information. We ran this test 150 times and recorded its success rate as 42%.

Furthermore, proper initial orientations can reduce the number of iterations for point cloud registration, i.e., the operation time. Thus, we compute the averaging time for registering scanned patches during the reconstruction of the incisor, premolar, and molar teeth with/without initial orientations as shown in Table 1. Generally, using the orientation provided by the gyro can highly accelerate the stitching process for at least 10 times.

### 6.4. Usability Study

We conducted a user study that evaluates the effectiveness of interactive visualization in improving scanning efficiency. We first recruited 5 volunteers for our studies from the National Taiwan University of Science and Technology and National Yang-Ming University. They had normal or corrected normal vision. Their ages ranged from 23 to 38 years old with a mean of 25.6; there were 5 males. The study was conducted using a PC with Intel i7-8700, 16 G memory, and Nvidia GTX 1080 Ti. All studies were conducted with an AOC i2757fm monitor at a resolution of 1920×1080, a brightness of 250 cd/m${}^{2}$, and a refresh rate of 60 Hz.

As visualization can assist the scanning process, we chose the isolated incisor, premolar, and molar as the target. We had two testing scenarios: scanning with/without interactive visualization. The user study was conducted as follows: (1) Participants were asked to complete a five-minute training session to familiarize themselves with the framework. (2) The instructor had the destined tooth and gave instructions including the scanning order and the procedural details. (3) Participants were asked to scan the isolated tooth in the designed order. (4) Our system interactively provided an immediate scanned result to participants for examination for the using scenario. (5) Steps 3 and 4 were repeated until the entire tooth was completely scanned; the instructor took all scanned data to check the success of reconstruction. (6) While failing, participants were asked to scan the tooth again. (7) The instructor chose another 2 teeth and asked participants to repeat steps 3–6 until they finished all three teeth. The order to scan three isolated teeth was predetermined as incisor, premolar, and molar. The order of whether to use visualization was chosen in a counterbalanced manner to eliminate order bias. During the study, we recorded how much time was used for executing scanning which excludes instructor’s examination and reconstruction time. Furthermore, while discussing with dentists, it is not acceptable to do the scanning process over 10 times. Thus, we set an upper bound of the total number of scanning operations as 11. Figure 7d–f shows the mean and standard deviation of the scanning time.

In this study, we sought to determine whether our tool helps the scanning operation. As differences among participants of different genders and ages—known as nuisance variables—could significantly contribute to error variances and thereby affect the final results, we used a randomized block factorization design (RBFD) [24] to employ a blocking procedure to assign the level of nuisance variations randomly to the experimental units to distribute the known and unsuspected variation sources among the units over the entire experiment in order to avoid effecting only one or a limited number of factors. In other words, the null hypothesis is that with/without interactive visualization has zero effect on scanning operation time. According to Table 9.5-1 of Kirk [24], we computed $F_{1,24}=493.9$ (*p* < $10^{-5}$) for the operational time. This reveals that the usage of visualization is a significant factor for the operational time. As shown, it is impossible for operators to successfully finish the dental reconstruction process within 11 times while our visualization framework can ensure the success of scanning and can largely reduce the scanning and reconstruction time. As shown in Figure 1, to finish scanning incisor, premolar, and molar teeth with our visualization would take an average of $1.51\times 10^{2}$, $1.75\times 10^{2}$, and $2.75\times 10^{2}$ s with standard deviations of 7.83, $4.43\times 10^{1}$, and $1.89\times 10^{1}$ s while it must take over $5.06\times 10^{2}$, $4.69\times 10^{2}$, and $1.52\times 10^{3}$ s with standard deviation of $4.60\times 10^{1}$, $5.22\times 10^{1}$, and $1.71\times 10^{2}$ s for other processes. Our visualization framework can ensure the success of the scanning process to largely reduce the number of operations and operational time. This can largely reduce the waiting time for patients and operators.

## 7. Conclusions

This work designs an interactive SSOCT-based gum-penetrative dental scanner to reconstruct the surface with a comparable precision to the state-of-art method. It can be applied frequently and repeatedly for possibly periodic inspection, but currently, it still has a few limitations, and there are a few future research directions. First, we only develop a conceptual protocol for demonstrating its possibilities, but the hardware structures and software algorithms may not be optimized. It is our future goal to optimize the hardware including having a better laser injector for better resolution and less noises, an efficient swept-source control mechanism for faster scanning and a larger caliber, and an on-board FFT chip. Currently, our goal aims at the ability of collecting plausible data using efficient and effective algorithms for visualization. To get precise reconstruction, it also requires optimizing the data-processing algorithms. Second, currently available SSOCT injectors can only emit a stripe of coherent infrared laser rays to limit its caliber. While observing the operational mechanism of intraoral scanners, they generally have a moving scanning mechanism. Therefore, it would be our future direction to have a line-stripe scanning mechanism incorporating better motion tracking and image stabilization for online video-like reconstruction. Third, although the scanning order and gyro’s initial orientation can improve the reconstruction robustness, sometimes flat regions can still cause the alignment failure. It would be interesting to incorporate a better motion-tracking mechanism for robustness. Fourth, the ability to acquire under-gum dental structures allows dentists to periodically examine their surfacing conditions. It would be interesting to extend its functionalities to examine sub-gum diseases such as dental cracks, caries, and periodontal diseases.

## Figures and Tables

**Table 1:** This shows the averaging stitching time in seconds with/without using the gyro information for the incisor, premolar, and molar teeth.

	Without (s)	With (s)	Acc.
Incisor	$3.70\times 10^{2}$	$3.51\times 10^{1}$	10.5
%midrule Premolar	$6.65\times 10^{2}$	$4.49\times 10^{1}$	14.8
Molar	$6.43\times 10^{2}$	$4.34\times 10^{1}$	14.8

## Abbreviations

The following abbreviations are used in this manuscript:

OCT	Optical Coherence Tomography
GPU	Graphics Processing Unit
4PCS	Super 4-Point-Congruent-Set
LIDAR	light detection and ranging
FFT	Fast Fourier Transform
CT	Computed Tomography
MRI	Magnetic Resonance Imaging
SSOCT	Swept-Source Optical Coherent Tomography
AFG	Arbitrary Function Generator
ADC	Analog-to-Digital Converter
TPS	Thin-Plate Spline
RANSAC	RANdom SAmple Consensus
DT	Transfer a slice of scanning data (DT)
Re	Optical Rectification (Re)
BD	Boundary Detection
